# The development and psychometric properties of the Prison Fellowship-Well-being Index (PF WBI)

**DOI:** 10.1108/IJOPH-03-2024-0009

**Published:** 2024-11-26

**Authors:** Michael Denhof, Rachel Crawley, Leigha Puckett, Jesse Wiese, Theresa Ferry

**Affiliations:** Department of Research and Innovation, Prison Fellowship, Lansdowne, Virginia, USA

**Keywords:** Offender health, Health promotion, Correctional health care, Prisoners, Prison staff, Health promoting prison

## Abstract

**Purpose:**

This paper aims to describe the development and validation of the Prison Fellowship Well-being index (PF-WBI), a new quantitative tool for assessing prisoner and staff well-being within prison cultures.

**Design/methodology/approach:**

The PF-WBI was developed through an iterative process of item creation, administration alongside established well-being measures and a series of data analyses. Data was collected from both staff and prisoners (*n* = 989) across four North Dakota prisons.

**Findings:**

Analysis supported a four-factor structure for the PF-WBI measuring motivation/self-esteem, relationships/community functioning, hope/mood and stress-related detriments. The PF-WBI demonstrated excellent internal consistency reliability, convergent validity with established well-being measures and criterion-related validity for both staff and prisoners. Measurement invariance across staff and prisoners was also confirmed.

**Originality/value:**

The PF-WBI offers a new and versatile tool for researchers and practitioners to assess staff and prisoner well-being in correctional settings. It can be used to evaluate prison cultures and the effectiveness of culture improvement efforts.

## Introduction

A growing body of research underscores the existence of a critical link between prison culture and the well-being of both staff and prisoners. Positive and value-driven cultures characterized by staff attributes of integrity, responsibility, respect for people’s inherent worth and valuing of community and productivity, have been found to have transformative potential for all constituents of prison culture. For example, such attributes and related dimensions (e.g. perceptions of authenticity, trust and safety) have repeatedly been found to correlate with a spectrum of indicators of staff and prisoner well-being.

Examples include increased perceptions of safety for staff (Palmen *et al.*, 2022) and prisoners (Bressington *et al.*, 2011); positive relations between prisoners (Kyprianides and Easterbrook, 2020); quality of staff–prisoner relationships (Bosma *et al.*, 2020); higher satisfaction, reduced burnout and turnover for staff (Lambert *et al.*, 2007; Rocheleau, 2014; Tracy, 2004); improved well-being or mental health of staff (Denhof *et al.*, 2014; Hayden and Huth, 2020) and prisoners (Beijersbergen *et al.*, 2013; Goomany and Dickinson, 2015; Gover *et al.*, 2000; Wooldredge, 1999); prosocial behavior of prisoners (Warren *et al.*, 2013a); rates of violent behavior and misconduct for prisoners (Bloom and Bradshaw, 2022; Bosma, *et al.*, 2020; Robinson *et al.*, 2018; Camp *et al.*, 2003; Long *et al.*, 2011); prisoner suicide (Huey and McNulty, 2005; Liebling, 2011; Slade and Forrester, 2015); and successful reentry (Auty and Liebling, 2020; Blagden and Perrin, 2016).

This evidence highlights the urgency of developing reliable and valid instruments to assess cultural factors vital to well-being within prison environments. Such tools are essential for identifying areas for improvement, guiding targeted interventions and monitoring the success of culture change initiatives (Beijersbergen *et al.*, 2013; Blagden and Perrin, 2016; Denhof *et al.*, 2024).

Complementing the assessment of core cultural values, an understanding of well-being outcomes amongst those working or living in prison provides important feedback on the impact of such values. Recognizing this need, Prison Fellowship (PF) has developed the Prison Fellowship Well-being Index (PF-WBI) [1] alongside the Prison Fellowship Prison Culture Assessment (PF-PCA) [2]. These tools offer a powerful combination for evaluating both the underlying values within prison culture and the associated well-being of staff and prisoners. Assessment results from these tools permit targeted interventions and the ability to track the relationship between culture and well-being over time. Although designed for complementary use, the PF-WBI and PF-PCA can also be used independently.

## Method

### Sample size and participants

***Staff participants*.** Staff participation was solicited facility-wide at four NorthDakota prison facilties using the state e-mail system or through internal announcements, at the discretion of the facility directors. In all cases participation was described in these communications as voluntary. The estimated total staff population across all four facilities was approximately 600. Staff were informed of the purpose, procedures, risks, benefits and voluntary nature of participation before consenting to a 90-min online survey/assessment battery. The battery contained the PF-WBI candidate items alongside items from several established instruments having conceptually similar or related assessment content. Prior to participation, electronic consent to an Institutional Review Board (IRB)-approved consent form was required. Approximately, 73% of prison staff ultimately participated (*n* = 435), which exceeded expectations. As shown in Table 1, participating staff were predominantly white, consisting of both males and females in near equal proportions, working within a variety of security levels and housing areas and having a range of years’ experience. The largest subgroup had five years or less of experience. Participating staff also reported holding diverse job roles spanning security, administration, programs, support services, management and other areas.

*Prisoner participants.* Lists of randomly-selected candidate prisoner participants were prepared by staff for use at individual prison facilities. Lists for each facility included an excess of potential participants (i.e. over-sampling) in anticipation of some degree of declined participation or unavailability, which might take place for a number of reasons (e.g. schedule issues; disinterest; prisoner-to-prisoner contact restrictions). A pool of approximately 1,300 prisoner participants across all four facilities were drawn upon.

Selected prisoners initially received letters informing them of voluntary participation opportunities on specified dates. On their assigned day, prisoners were contacted and either called out or escorted to receive a live participation offer which included information on an informed consent requirement [3]. Participation offers and actual participation took place in a group format within dedicated classrooms or other large open areas. Spaces included tables and chairs and enough room for reasonably private participation by individuals. Paper assessment materials, pencils and snacks were provided. Participants were instructed to raise their hand with any questions that might arise during participation (e.g. misunderstanding of a question or word’s meaning). Researchers assisted individuals as discreetly as possible, and as needed. Non-English-speaking or mentally impaired individuals were dismissed if there was an inability to read or comprehend [4].

Researchers initially observed poor responsiveness to participation offers, which motivated researchers to permit participation of word-of-mouth volunteers. Ultimately, an estimated 70% of the final participant sample were randomly selected with 30% being volunteers (*n* = 594) [5]. As shown in Table 2, over 90% of participating prisoners were male, of diverse age and had been incarcerated for a duration of anywhere from three months to 20 years. Participating prisoners demonstrated a variety of ethnic/cultural affiliations, came from different security levels and resided in a range of in-prison housing areas.

## Measurement instruments

An array of five well-established and one more recently developed psychometric assessment instruments were selected for concurrent administration alongside the PF-WBI. Compiling response data from this standard battery provided opportunity for a substantive evaluation of the PF-WBI’s convergent and criterion-related validity. This was accomplished through correlational analysis procedures and examination of patterns and magnitudes of relation between participants’ scores on the PF-WBI and their observed standing on the various other instruments that were designed to measure similar or related content.

### Depression, Anxiety, and Stress Scales (DASS-21)

The widely used Depression, Anxiety and Stress Scales (DASS-21) from Lovibond and Lovibond (1995) constitutes a 21-item questionnaire requiring respondents to rate the applicability of a series of affective statements based upon intensity of symptoms experienced over the past week. Using a four-point response scale, the DASS-21 has been found to demonstrate adequate levels of internal consistency reliability, ranging from 0.82 to 0.93 (Henry and Crawford, 2005). It has exhibited favorable performance across translations, including both confirmed cross-cultural equivalence and measurement stability across international populations (Bibi *et al.*, 2020; Dreyer *et al.*, 2019; Tonsing, 2014). Previously published findings further support the tool’s discriminant validity at the level of individual items (Antony *et al.*, 1998) with aggregated scores occasionally serving as a barometer of general psychological distress (Henry and Crawford, 2005).

### Brief Sense of Community Scale (BSCS)

The Brief Sense of Community Scale from Peterson *et al.* (2008) uses eight questionnaire items coupled with a five-point response scale to assess facets of perceived belonging within a particular social collective. It is based upon foundational theoretical tenets regarding common group experiences, affiliation and interpersonal cohesion (McMillan and Chavis, 1986). In addition to exhibiting excellent internal reliability estimates of around *α* = 0.92 in initial validation work, subsequent research has affirmed stable utility of the tool across diverse cultures and settings. This includes successful applications gauging psychological sense of community within various universities, neighborhood, health care, religious, correctional and other community formations (Balboni *et al.*, 2018; Cardenas *et al.*, 2021; Yu *et al.*, 2022).

### Responsibility Questionnaire (RQ)

The responsibility questionnaire from Arslan and Wong (2022) constitutes an efficient eight-item tool using a five-point Likert response scale designed to quantitatively index variability across the dual domains of personal and social responsibility dispositions in adult populations. Increased scores along these subscales have been found to demonstrate expected correlations with related psychosocial criteria such as life satisfaction, conscientious, characterological tendencies toward order, detail-focus and general psychological distress stemming from inadequacy in meeting situational demands and obligations (Arslan and Wong, 2022).

### Big Five Inventory (BFI)

The Big Five Inventory (BFI) is a well-established measure of normative personality traits with demonstrated evidence of stable cross-cultural performance, including across translated language versions (Ubbiali *et al.*, 2013; Leung *et al.*, 2012). Grounded in the five-factor model of dispositional attributes, this comprehensive assessment consists of 44 statements requiring participants to rate self-applicability along intensity continuums spanning five primary personality spectra contrasting extraversion-introversion, antagonism-agreeableness, lack of direction-conscientiousness, neuroticism-emotional stability and closedness–openness traits. In light of the conceptual relevance of prison culture values (e.g. community, affirmation, restoration) to well-being, the current study used only the BFI's Agreeableness and Conscientiousness subscale scores.

### Flourishing scale

The Flourishing Scale (FS) (Diener *et al.*, 2009) is an efficient eight-item index using a seven-point Likert response format designed to subjectively quantify variability across multiple domains of positive psychological functioning. Areas include perceived success in handling relationships, positive self-appraisal, sense of life purpose and optimism. Considered a barometer of overarching psychological resources and strengths, aggregate scores from the measure have exhibited adequate reliability and substantial correlations with complementary indicators of health and wellness when implemented across cultures (Hone *et al.*, 2014; Silva and Caetano, 2013; Sumi, 2014).

### Prison Fellowship-Prison Culture Assessment (PF-PCA)

The PF-PCA is a rigorously developed five-scale, 44-item, quantitative questionnaire that uses a five-point response format with the following anchors: False, Slightly True, Fairly True, Mostly True, Very True. The instrument was designed to tap the status of five key value dimensions within prison staff, as a reflection of the social culture. Measured dimensions are defined as the demonstration and valuing of community, affirmation, productivity, restoration and integrity/responsibility (Denhof *et al.*, 2024) by prison staff. Data is collected from both staff and prisoners for comprehensive and inclusive results. Prison staff are understood to have the onus of responsibility for the form and quality [6] of prison culture, as well as its consequences.

Grounded in PF’s Good Citizenship Model® (GCM; Wiese *et al.*, 2024), which proposes development of key values as a basis for positive identity development, well-being and flourishing, the PF-PCA’s development process produced strong evidence of reliability (*α* > 0.90) across each of five subscales. Concurrent and convergent forms of validity haev been established through substantive correlations with a variety of relevant and related variables. The instrument is unique among available prison culture or climate assessment instruments in its ability to capture and quantitatively compare the perspectives of both staff and prisoners using identicial assessment items. Evidence of configural and metric validity have been established and scalar-level invariance tentatively established, across staff and prisoner subpopulations.

## Planned analyses

### Structural analyses

The total data set was to be randomly split into calibration and validation halves for purposes of cross-validation (Byrne, 2016). Using this approach, if an identified factor structure based upon calibration data was subsequently found to be recoverable in a second set of validation data, this would constitute evidence of factor structure stability. Principal components analysis (PCA) would be used to analyze the calibration data. Confirmatory factor analysis (CFA) would be used to more rigorously confirm the factor structure identified through PCA.

### Principal components analysis

PCA would be performed with a primary interest in exploring the dimensionality of candidate well-being items and eliminating items with poor loadings or insufficiently distinctive loadings in the case of multiple factors. The intent is to capture well-being with a broad net and to potentially also capture multiple well-being subdimensions through factors – reflecting, for example, social, personal, emotional and/or aspirational aspects.

### Confirmatory factor analysis

CFA was to be subsequently performed upon the PCA-identified factor structure by testing the model’s fit to the data when constraining particular items to load only on their conceptually appropriate factors. This analysis would rely upon data from the randomly selected calibration half of the sample and would provide results bearing on the recoverability of the specified factor structure.

### Factorial invariance assessment

The PF-WBI factor structure, in turn, would be assessed for configural, metric and scalar levels of invariance across prison staff and prisoner subgroups. Evidence of invariance is useful as it helps inform the extent to which an assessment instrument operates equivalently across different groups. Knowing the extent of invariance bears, for example, on how directly group mean scores for different populations can be compared (e.g. roughly versus point-for-point). Each of the three levels of invariance was assessed through a common approach: comparing the fit of a series of nested multigroup CFA models and examining fit discrepancies between successive models that are distinguished through specification of increasingly stringent equality constraints (Byrne, 2016; Collier, 2020; Kline, 2016). The magnitude of differences in fit index values from one nested CFA model to the next provides the basis for determining invariance (Kline, 2016). Compartive fit index (CFI) and root mean square error of approximation (RMSEA) difference criteria of CFI ≤ 0.01 and/or RMSEA ≤ 0.015 are commonly taken as evidence of invariance.

The first type of invariance to be assessed is configural invariance. Configural invariance requires that individual items load upon, and only upon, their intended factors – thereby requiring an appropriate pattern of loadings. The second type of invariance to be assessed is metric invariance, which requires that items load exclusively upon their intended factors but also that the magnitudes of such loadings are invariant across groups. Historically, evidence supporting configural and metric invariance has been considered sufficient for many assessment instruments and purposes. Nevertheless, additional levels of invariance, including the scalar level, are increasingly being pursued by instrument developers (Putnick and Bornstein, 2016). This is particularly true when scores are being interpreted or compared in a way that might have serious consequences for an assessed individual or group. Scalar invariance requires the establishment of equal intercepts in addition to configural and metric invariance.

### Convergent and criterion validity evaluation

Foundational convergent and criterion-related validity would be assessed through examination of correlations between the PF-WBI’s overall score [7] and 12 scale/subscale scores from six established multiscale instruments that were administered concurrently. The established instruments were chosen due to having content that was expected to be either conceptually similar (i.e. bearing on convergent validity) or related to PF-WBI content (i.e. bearing on criterion-related validity).

Correlations between the PF-WBI overall score and the FS primarily, as well as the DASS-21’s overall scale and subscale scores secondarily, were expected to be substantive and representative of convergent validity. This was expected because of these measures’ conceptual similarity to the construct of well-being. Relations between the PF-WBI and each of the remaining concurrently administered measures were also expected to be substantive given existing research findings bearing on relationships between prison culture form/quality and well-being level, among other prison outcome variables. The remaining correlations provided opportunity to assess for criterion-related validity. Variables reflecting the relative presence of socially functional and value-laden character dimensions such as are measured by the PF-PCA and related measures (e.g. sense of community, conscientiousness, responsibility, etc.) were expected to influence the well-being of both staff and prisoners.

### Additional psychometric property information

Following factor analyses, PF-WBI scale and subscale reliability and intercorrelations would be calculated and summarized. Scale reliability would be estimated using Cronbach’s alpha (*α*) applied to subsets of items pertaining to WBI scales and potential subscales. Pearson intercorrelations among subscales would be calculated and summarized for descriptive purposes and for potential conceptual or theoretical implications. Mean factor loadings and squared multiple correlations (SMCs) would also be calculated for item subsets pertaining to each of the PF-WBI’s subscales. SMCs bear on how well targeted content is captured by constituent items.

### Data preparation

An initial pool of *n* = 1,029 participants (*n* = 435 staff, *n* = 594 prisoners) provided data on a 231-item assessment battery covering seven instruments and 19 subscales. The extent of missing data due to some individuals not finishing the questionnaire battery varied for staff (12.6%) and prisoner (2%) subgroups. This was likely due to survey length [8] and/or the fact that staff were not directly monitored during participation as prisoners were. Missing data, exclusive of discontinuation cases, constituted only 4% and was considered missing at random (MAR). Analysis-specific approaches to addressing missing data were used. In relation to the performance of PCA, correlation significance testing, and assessing scale reliability – SPSS’ listwise, case wise or means imputation options were used, as needed/appropriate. In relation to the conduct of CFA or factorial invariance assessment – AMOS’ full information maximum likelihood estimation was relied upon.

Variables were found to exhibit minimal departures from normality (skewness <2, kurtosis <5), safeguarding analytical performance (Muthén and Kaplan, 1985, 1992). The robust maximum likelihood estimation method further mitigated concerns. To address multivariate outliers – potentially indicating careless responses – we used Mahalanobis distance. This approach, justifiable given the unique challenges of data collection within prison settings, led to the removal of 4% of cases, resulting in a final sample of *n* = 989 for subsequent analyses.

## Results

### Principal components analysis

Analysis revealed that several facets of well-being were unique enough to form some distinctive factors. A relatively high minimum factor loading criteria of 0.5 was used, to promote the creation of robust factors. A series of PCA analyses were run while varying analysis options and examining resulting output (e.g. targeted number of factors, rotation type). Analyses ended with identification of a conceptually clear four-factor solution with no secondary loadings ≥0.3. Given a more rigorous CFA would be subsequently applied, and to conserve space, only details of the CFA are presented.

### Confirmatory factor analysis

The four-factor structure identified within the calibration sample was reassessed using CFA and the validation sample data. The specified model was found to be recoverable. To follow, it was confirmed that assessment results – bearing on the fit of the four-factor measurement model to the calibration, validation and aggregate/total samples – were found to be virtually identical. Therefore, only results from the total sample are detailed, as they were expected to provide the most representative results. Table 3 shows the CFA estimates of overall model fit that were estimated, based upon various established indices of model fit: the CFI, the incremental fit index (IFI), the Tucker-Lewis Index (TLI), RMSEA and the chi-square to degrees of freedom ratio (*χ*^2^/*df)*. All estimates were found to support adequate model fit based on commonly accepted criteria for index value interpretation (e.g. CFI ≥ 0.90 or 0.95; IFI ≥ 0.90 or 0.95; TLI ≥ 0.90 or 0.95; RMSEA ≤ 0.08 or 0.06; χ^2^/*df* ≤ 2 or 5). These findings support both the PF-WBI’s conceptual structure and its ability to distinctively measure each of four subtypes of well-being.

**Figure 1:**
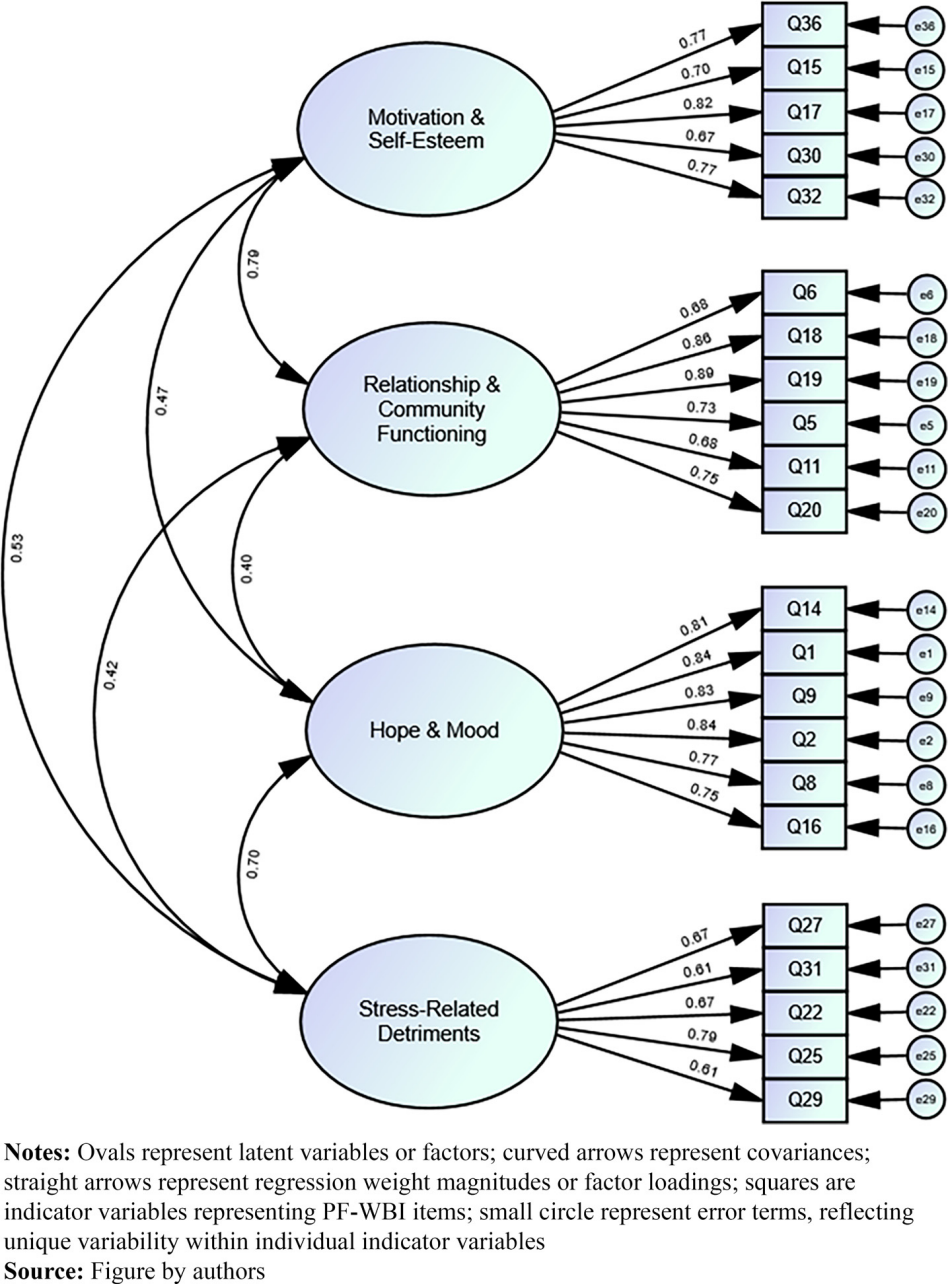
Prison Fellowship–Well-being Index (PF-WBI) four-factor measurement model

Figure 1 shows the measurement model visually as a path diagram, including standardized estimates for factor covariances and factor loadings. For clarity, error terms are not shown, and there was no specification of correlated errors in the CFA. In addition, Table 4 shows mean standardized factor loadings and mean SMCs pertaining to the item subsets defining each factor. The mean factor loadings were found to be robust, averaging 0.75 and ranging from 0.67 to 0.81. All individual item loadings were found to be statistically significant at *p* < 0.001. SMCs were found to average 0.57 and ranged from 0.46 to 0.65.

### Factorial invariance assessment

Table 5 summarizes the results of the nested multigroup CFA model comparisons that were conducted to verify invariance across the two prison culture subpopulations of staff and prisoners. In this context, the magnitude of differences in fit index values from one nested CFA model to the next provides the basis for determining invariance (Kline, 2016). CFA and RMSEA differences, denoted in the table as ΔCFI and ΔRMSEA, are commonly employed for this purpose, where fit discrepancies of CFI ≤ 0.01 and RMSEA ≤ 0.015 supportive invariance. As indicated in Table 5, configural, metric and scalar levels of invariance were all supported, confirming the applicability of the PF-WBI for use with multiple subgroups of prison culture.

## Prison Fellowship-Well-being Index scale and subscale definitions

After confirming the stability of the four-factor measurement model using EFA and CFA techniques, and finalization of scale item content, PF-WBI scale and subscale definitions were prepared. Tables 6 and 7 provide definitions as well as suggested guidelines for score interpretation. The table’s contents are based upon rationally derived cut-points and interpretive categories.

## Convergent and criterion validity evaluation

**Figure 2:**
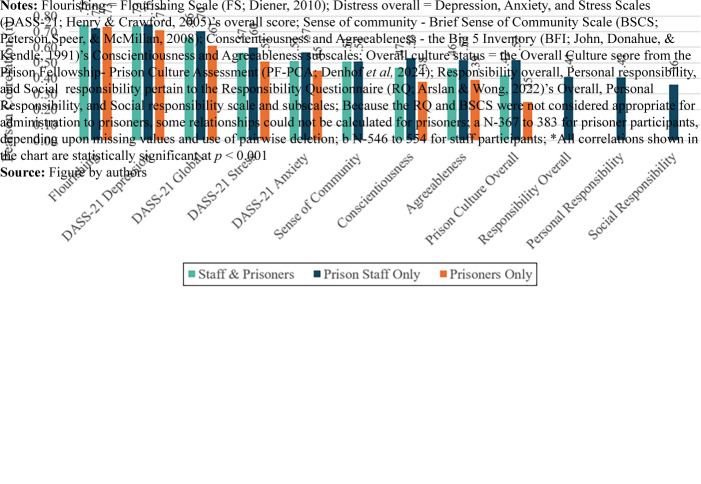
Correlations between the PF-Well-being Index (PF-WBI) scale and a range of similar constructs and related variables

The PF-WBI overall score is defined as the average of all responses to the PF-WBI’s 22 individual items. Given the large number of variables involved, relationships between the PF-WBI’s overall culture score and each of a spectrum of concurrently administered measures were charted (Figure 2). This focus on the PF-WBI’s overall score in relation to other variables permitted a more parsimonious description of fundamental relationships bearing on validity. Separate correlations are shown for calculations based upon collapsed staff and prisoner data, staff-only data and prisoner-only data.

As can be observed in Figure 2, virtually all measured relationships were substantive. The strongest correlation magnitudes were demonstrated for relationships between the PF-WBI Overall Well-being score and scores from: the FS and DASS-21's Depression, Overall Distress, Stress and Anxiety subscales. Correlation magnitudes were substantive regardless of whether calculations were based on aggregated data from staff and prisoners or from independent subsets of staff or prisoners. All correlations were found to be statistically significant at *p* < 0.001. These relationships strongly support the convergent validity of the PF-WBI.

Observed correlations between the PF-WBI Overall Well-being score and measures of sense of community, prison culture quality, conscientiousness and agreeableness scales as well as overall, personal and social forms of responsibility were substantial and statistically significant (*p* < 0.001). One correlation, between the PF-WBI Overall Well-being score and PF-PCA Overall Culture score, was noticeably lower in magnitude for prisoners (*r* = 0.25) than for prison staff (*r* = 0.52).

## Prison Fellowship‐Well‐being Index (PF‐WBI) reliability

Alpha was calculated for each of the PF-WBI’s five subscales. Alpha values, along with means and standard deviations can be found in Table 8. Calculations based solely upon staff data showed an alpha value of 0.93 for the PF-WBI overall scale and values ranging from 0.83 to 0.93 for the four PF-WBI subscales. Calculations based upon prisoner data showed an alpha value of 0.92 for the PF-WBI overall scale and values ranging from 0.78 to 0.91 for the subscales. Collectively, the observed alpha values suggest excellent reliability of all PF-WBI scales and subscales when applied to staff and/or prisoners.

Not surprisingly, mean PF-WBI Overall Well-being and subscale score magnitudes were in general found to be somewhat lower for prisoners than for staff. This was found to be true for all scale and subscale means reported in Table 8.

## Prison Fellowship-Well-being Index subscale intercorrelations

Pearson correlations between all PF-WBI subscale scores were calculated and can be observed in Table 9. PF-WBI subscale scores were calculated by averaging the four-point scaled responses to each subscale’s constituent items. All subscale scores were found to be correlated with each other to a substantive degree. In addition, all correlations were found to be statistically significant with magnitudes ranging from 0.33 to 0.69.

## Discussion

This development project provides foundational evidence supporting the reliability and validity of the newly developed PF-WBI as a robust measure of well-being within prison settings. Our results strongly support a four-factor structure, revealing conceptually distinct and practical subdimensions of well-being for both prison staff and prisoners: Motivation/Self-Esteem, Relationship/Community Functioning, Hope/Mood and Stress-related Detriments. The PF-WBI overall scale, as well as each subscale, demonstrate excellent internal consistency reliability across both populations. This finding permits correctional leadership and researchers to use the PF-WBI with confidence in its ability to generate stable scores for such purposes as measuring the impact of prison culture improvement efforts or of other programs expected to promote well-being. The need for effective measurement of well-being in correctional settings has been highlighted in recent studies, pertaining to both staff (National Institute of Justice, 2023) and incarcerated people (Criss and John, 2023).

To establish convergent validity, we examined the relationships between the PF-WBI Overall Well-being score and several conceptually similar measures, including the FS and the Depression, Anxiety and Stress Subscales (DASS-21). Relationships were measured for the total sample and for staff and prisoner subgroups individually. As expected, strong correlations emerged for both subgroups, underscoring the PF-WBI’s alignment with existing scales that can be seen as fairly direct indicators of well-being or its absence. This general finding provides substantial evidence to support the view that the PF-WBI captures well-being in a manner similar to several established measurement scales. Although the established measures used for validity assessment were not developed specifically for use with corrections populations, the universal nature of the construct of well-being (Diener *et al.*, 2018) nevertheless facilitated confirmation of substantive relationships.

Evidence of criterion validity was established through the PF-WBI Overall Well-being score’s measured relationships with several related variables: sense of community, the character attributes of conscientiousness and agreeableness, various forms of responsibility and an overall measure of prison culture – as it is constituted though prison staff values and behavior on the job. One notable observation was that the correlation between the PF-WBI overall score and PF-PCA overall score was lower in magnitude when calculated for prisoner subgroup (*r* = 0.25) versus the prison staff subgroup (*r* = 0.52). It would seem plausible that this difference is related, at least in part, to the prisoner population having more mental health issues independent of prison culture quality than do prison staff, on average. It might also be due, to some extent, to how prisoner responses to PF-PCA items are necessarily more inferential and deductive for prisoner respondents than for staff respondents, given how the PF-PCA’s content is focused primarily upon prison staff values and behavior.

These findings, reflecting criterion-related validity, are consistent with a growing literature bearing on an important broad stroke relationship between prison culture’s form/quality and the well-being of people who work or live in prisons. Adding criterion-related validity evidence to the aforementioned convergent validity evidence further strengthens support for the overall validity of the PF-WBI for its intended purposes.

Configural, metric and scalar levels of measurement invariance were assessed to confirm whether the PF-WBI performed invariantly across staff and prisoner populations. Findings indicated that all three levels of invariance were supported, demonstrating a strong justification for aggregating scores from all members of prison culture for culture-level well-being analysis purposes and for potential comparisons of scores across staff and prisoner subgroups.

The internal consistency reliability of the WBI overall and subscale scores were calculated, for the total sample in aggregate as well as for staff and prisons separately. Results generally ranged from *α* = 0.8 to 0.9, exceeding the commonly recommended criterion of 0.7 or higher, and where higher scores reflect higher reliability (Netemeyer *et al.*, 2003). This finding confirms the ability of corrections leadership, prison researchers or program providers to obtain stable and consistent measurements when implementing the PF-WBI for well-being assessment. Mean factor loadings for subscales were found to be robust and statistically significant. Mean SMCs fell within a range suggesting latent constructs that are reasonably well-defined by their constituent items (Tabachnick and Fidell, 2001).

In regard to the magnitude of PF-WBI scale and subscale scores, prisoners demonstrated lower scores than staff overall and in relation to each PF-WBI subscale. This pattern was not surprising. Although prison work is inherently difficult and stressful, prisoners nevertheless can be expected to experience even lower well-being levels than staff, on average. Prisoners are, after all, incarcerated against their will, subject to an extreme power differential during incarceration, and must cope with minimally comfortable conditions of confinement. It should be noted, however, that mental health disorders (Denhof and Spinaris, 2013; Fazel and Danesh, 2002; Obidoa *et al.*, 2011) and suicide risk (Fazel *et al.*, 2011; Stack and Tsoudis, 1997) have been found to be elevated for both staff and prisoners relative to members of the public or general population. Although the stress of incarceration has long been acknowledged, prison work also involves exposure to a variety of stressors (Denhof *et al.*, 2014) that can and often do have substantial consequences upon staff well-being, health and functioning.

Results showed the presence of substantive PF-WBI subscale intercorrelations. This is unsurprising given that the candidate PF-WBI items were all intended to capture some aspect of well-being, broadly conceived. The observed correlations also align with theoretical expectations. For example, an individual’s sense of motivation and self-esteem has been found to promote healthier social connections and foster positive relationships (Crocker and Park, 2004). Simultaneously, robust social support systems have been found to play a critical role in building self-esteem and enhancing emotional well-being (Cohen and Wills, 1985). The positive correlation between hope/mood and motivation/self-esteem also makes sense since having a sense of purpose and agency reinforces positive outlook (Snyder *et al.*, 1991). Conversely, the negative correlation between stress-related detriments and the other well-being dimensions is well-documented, as chronic stress has been found to impair mood, damage relationships and erode self-confidence (Slavich and Irwin, 2014).

Taken collectively, the above-described validity evidence and psychometric properties provide a strong foundation of support for the reliability, validity and measurement performance of the PF-WBI, which was developed specifically and from the ground up for use in correctional settings. Further understanding of the PF-WBI and its performance characteristics might come through future studies designed to assess the ability of PF-WBI scores to predict routinely collected prison outcome measures cross-sectionally or longitudinally (e.g. institutional misconducts or post-release outcomes). Potential lines of inquiry might include the PF-WBI’s ability to predict rehabilitative program performance or its relationship to staff turnover rates.

PF’s commitment to ongoing PF-WBI and PF-PCA development is evident in its data accumulation efforts to date. This data collection tool place primarily through its Warden Exchange® (WE) program (Prison Fellowship, 2024) activities. WE seeks to optimize a variety of prison outcomes for staff and prisoners alike, through transformative leadership training and prison culture improvement planning and efforts. WE-based data collection has resulted in an expanded collection of anonymized PF-PCA and PF-WBI data from across the USA that permits more stable and generalizable baselines to facilitate score interpretations, such as through regional and national averages. In addition, invariance assessments across gender and ethnic subgroups are planned, to further validate the tool’s equitable performance when applied to people of varying demographics.

Finally, PF’s recent development of a secure web-based dashboard [9] can empower partner organizations to efficiently monitor both well-being and culture quality levels using PF-WBI and PF-PCA scores. The web-based system permits automated cross-sectional and longitudinal charting of scores reflecting culture and well-being status, as well as automated report generation that includes score interpretation and data-driven recommendations for culture improvement efforts. The ability to concurrently observe both culture status and well-being scores over time helps reinforce for corrections leaders an established and observable relationship between culture quality and well-being levels. Equipped with dashboard and automated reporting tools, it is hoped that corrections leaders will benefit from an increased sense of control over both the form of their culture and, in turn, the well-being of their staff. Ultimately, such efforts may positively impact not only well-being but also other critical correctional outcomes, such as suicide rates, post-release success for prisoners, staff job satisfaction and longevity, and more broadly, community safety.

## Limitations and future directions

Although this project offers strong foundational support for the PF-WBI, there are limitations to consider. Prison environments inherently pose challenges to research efforts, such as in relation to recruitment and retention of participants. This is due to factors such as heightened stress, complex safety protocols, logistical constraints and understandable mistrust or concerns about confidentiality – among both prisoners and staff. These circumstances, present in our project, may have degraded, at least to some degree, the purity of random selection. As such, generalization of findings technically warrants at least some degree of caution. Despite the well-known difficulty, in general, of obtaining pristine sample data from prisoners and staff in correctional settings, the conduct of research in these settings remains paramount. Replication of studies and ongoing data collection provide a remedy for mitigating the impact of sampling flaws and reinforcing the accuracy of findings over time.

Another noteworthy point is that the initial development of the PF-WBI used data from a prison system within a single state. Although the universally human nature of well-being measures can be expected to minimize concerns about geographic specificity, time and replication of findings across states and regions nevertheless can be understood as a path to confirming and documenting generalizability and applicability.

## Conclusion

This PF-WBI development project establishes a strong foundation of evidence for a new psychometrically sound instrument for assessing well-being specifically within prison environments. Rigorous analysis across staff and prisoner populations demonstrated a stable four-factor structure, excellent reliability and multiple forms of convergent and criterion-related validity evidence. The PF-WBI effectively measures practical and relevant dimensions of well-being as an important outcome in its own right but also as a fundamental indicator of success in relation to prison program and prison culture improvement efforts.

Considering the established relationship between staff well-being and such important prison outcomes as staff retention, performance and absenteeism, as well as the link between prisoner well-being and rehabilitative outcomes, the PF-WBI offers a valuable new instrument for correctional systems. By utilizing PF-WBI scores, prisons can track well-being levels, guide data-driven decision-making and target specific cultural elements for improvement. This focus on culture as a leverage point aligns with growing evidence of its influence across a range of key prison outcomes.

The PF-WBI’s consistent performance across staff and prisoners supports its use for assessing entire prison wellness levels and/or monitoring staff and prisoner subgroups over time. Integrating the PF-WBI results into dashboarding systems extends the ability of correctional leadership to conveniently and systematically monitor well-being levels for all of their culture’s constituents. This empowers them to better support positive outcomes for staff, prisoners and the larger community. Continued research and implementation is expected to further solidify the PF-WBI’s distinctively useful role.

## Acknowledgements

The authors wish to thank the North Dakota Department of Corrections and Rehabilitation (NDDCR) for their repeated dedication of time and resources to assist this project. The authors received extensive hands on support from a range of prison wardens, deputy wardens and a variety of supporting staff that are too numerous to mention. The authors would like to mention, in particular, NDDCR’s David Krabbenhoft, Director of Administration at the NDDCR, Colby Braun, Interim Director of Administration, and Steven Foster, Deputy Warden of Programs at the North Dakota State Penitentiary (NDSP).

### Declaration of conflicting interests

Conflicts of interest: No conflicts of interest were identified or anticipated. Although Prison Fellowship, a 501(c)(3) non-profit organization, anticipates promoting and facilitating PF-WBI use by interested correctional organizations and leadership, this is not a for-profit endeavor. Prison staff, prisoners, prison programs, and entire organizational cultures stand to potentially benefit from the availability of the new PF-WBI instrument. All listed authors are employed by Prison Fellowship, but the PF-WBI project and product provide no mechanisms for financial gain.

## Figures and Tables

**Table 1:** Participant demographic characteristics – prison staff

Demographic	Valid (%)	Demographic	Valid (%)
*Security level*		*Corrections work (yrs.)*	
Maximum/super	41.4	0–5	44.2
Medium	32.1	6–10	18.2
Minimum	14.2	11–15	10.5
Multiple	10.9	16–20	8.9
		>20	18.2
*Ethnic group*			
White	84.7	*Job category*	
Black/African American	5.8	Security/custody	59.5
Native American	2.1	Non-security/custody	40.5
Latino/a	1.6		
Asian	0.3	*Job role*	
Mixed/multiple	2.9	Security	50.0
Other	2.6	Administrative	13.2
		Programs	11.1
*Gender*		Support	9.2
Male	52.9	Other	16.6
Female	45.8		
Other	1.3	*Specific job titles*	
		corrections officer	41.6
*Age range (years)*		Manager/supervisor	18.9
18–29	15.8	Mental health provider	5.8
30–41	34.5	Medical staff	5.8
42–53	28.7	Mid-level manager	3.9
54–65	18.7	Clerical	3.2
		Executive staff	1.6
		Other	16.3

**Notes:** ^a^Percentages are reported after excluding missing cases, so that percentages still add up to 100%, save rounding error;

^b^ *n* = 435

**Source:** Table by authors

**Table 2:** Participant demographic characteristics – prisoners

Demographic	Valid (%)	Demographic	Valid (%)
*Security level*		*Age range (years)*	
Maximum/super	21.8		
Medium	38.1	30–41	39.6
Minimum	31.5	42–53	24.1
multiple levels	7.1	54–65	9.5
Other	1.9		
*Ethnic group*		*Total years incarcerated*	
White	48.7	0–5	43.6
Black/African American	10.5	6–10	28.1
Native American	19.9	11–15	12.8
Latino/a	3.9	16–20	9.0
Asian	0.4	20+	6.6
Mixed/multiple	13.2		
Other	3.5		
*Gender*			
Male	92.8		
Female	5.5		
Other	1.7		

**Notes:** ^a^Demographic variable percentages shown are exclusive of missing values, which amounted to 2.2%–4.2% per demographic;

^b^ *n* = 594

**Source:** Table by authors

**Table 3:** Goodness-of-fit statistics for the four-factor PF-WBI measurement model

CFI	IFI	TLI	RMSEA	RMSEA 90% CI	χ*^2^*/*df*
0.95	0.95	0.94	0.053	0.051–0.059	4.03

**Notes:** CFI = comparative fit index (Bentler, 1990); IFI = incremental fit index (Bollen, 1989); TLI = Tucker–Lewis index (Tucker and Lewis, 1973); RMSEA = root mean square error of approximation (Browne and Cudeck, 1989); ^a^*n* = 989 after multivariate outlier removal

**Source:** Table by authors

**Table 4:** Mean regression weights and squared multiple correlations (SMCs)

PF-WBI factors	Mean factor loadings	Mean SMCs	# Items
Motiv. and Self-Esteem	0.75^*^	0.56	5
Relationship and C.F.	0.77^*^	0.59	6
Hope and Mood	0.81^*^	0.65	6
Stress-related Detriments	0.67^*^	0.46	5

**Notes:** ^a^*n* = 989 after multivariate outlier removal.

*Statistically significant at *p* < 0.001

**Source:** Table by authors

**Table 5:** CFI and RMSEA difference tests of nested invariance models

Model	χ^*2*^	*df*	Model comparison	ΔCFI	ΔRMSEA
Model 1 (configural/unconstrained)	3,563.22	1,384	--	--	--
Model 2 (metric/equal factor loadings)	3,686.77	1,418	2 vs 1	0.002	0.001
Model 3 (scalar/equal intercepts)	4,347.45	1,457	3 vs 2	0.007	0.002

**Note:** ΔCFI = CFI difference; ΔRMSEA = RMSEA difference

**Source:** Table by authors

**Table 6:** PF-WBI Overall Well-being and subscale definitions

Scale	Definition
Overall Well-being	The PF-WBI’s overall well-being scale score is an aggregate score, comprising several subsets of items representing different facets of well-being, as defined below. Its items capture well-being wholistically
Relationship and Social/Community Functioning	The Relationship and Social/Community functioning subscale captures the extent to which individuals or groups: trust the people that they interact with; feel supported by the people around them; feel encouraged by the good quality of their relationships; feel energized by their relationships; feel appreciation for the people around them; and/or feel comforted by support from friends, family and/or significant others
Hope and Mood	The Hope and Mood subscale captures the extent to which individuals or groups: feel their life is worthwhile; have devalued their life as a function of repeated negative life experiences; feel hopeless about the prospect of improving their life; feel worthless as a person; anticipate a happy future; and/or expect that other people would not be sad about one’s death
Stress Detriments	The Stress Detriments subscale captures the extent to which individuals or groups: are experiencing recent difficulty concentrating; experience psychological reactions when reminded of unpleasant events; have trouble relaxing; feel disconnected from others; and/or are noticing a recent decrease in memory functioning
Motivation and Self-Esteem	The Motivation and Self-Esteem subscale captures the extent to which individuals or groups: feel proud of themselves; are experiencing a high level of self-esteem; have felt enthusiastic lately; are feeling fulfilled in their life; and/or feel satisfied with their life in an overall way

**Note:** Some assessment item content is reverse-worded and reverse-scored

**Source:** Table by authors

**Table 7:** PF-WBI score interpretation key

PF-WBI scale score	Interpretation
>3.5	High
>3.0	Moderate
>2.5	Minimally adequate
≤2.5	Low

**Source:** Table by authors

**Table 8:** PF-WBI Overall Well-being and subscale characteristics for staff, prisoners and both

PF-WBI scales/subscales	*M* staff/prisoners/all	*SD* staff/prisoners/all	Cronbach’s alpha (*α*) staff/prisoners/all	No. of items
Overall Well-being	3.25/2.95/3.07	0.52/0.58/0.57	0.93/0.92/0.92	22
Motivation and Self-Esteem	2.89/2.57/2.70	0.74/0.80/0.79	0.89/0.84/0.86	5
Relationship and Comm. Functioning	3.07/2.77/2.90	0.70/0.80/0.75	0.90/0.88/0.89	6
Hope and Mood	3.76/3.48/3.59	0.55/0.72/0.67	0.93/0.91/0.92	6
Stress-related Detriments	3.23/2.89/3.03	0.71/0.75/0.75	0.83/0.78/0.81	5

**Source:** Table by authors

**Table 9:** Prison Fellowship – Well-being Index (PF-WBI) subscale correlations

	Motiv. and Self-Esteem	Relationship and C.F.	Hope and Mood	Stress-related Detriments
Motiv. and Self-Esteem	1	0.69**	0.42**	0.43**
Relationship and C.F.	0.69**	1	0.38**	0.33**
Hope and Mood	0.42**	0.38**	1	0.59**
Stress-related Detriments	0.43*	0.33**	0.59**	1

**Notes:** *Statistical significance at *p* < 0.01; **statistical significance at *p* < 0.001

**Source:** Table by authors

## Notes

1. This PF-WBI development project and each of its phases and components were approved by an IRB.

2. A manuscript detailing the PF-PCA development project has been submitted for publication.

3. At the time the voluntary participation offer was verbally presented to potential participants in a group format, IRB-approved consent forms were also handed out and explained. Anonymous participation was promoted by collecting signed consent forms in a separate bin from completed questionnaires – which required no identifying information.

4. To ensure compatibility of the WBI’s readability with both staff and prisoners, a pilot phase of this project was conducted prior to the described administration phase. It entailed administering the candidate WBI assessment items to 20 randomly selected prisoners from four facilities with the instruction that they bring to the attending researchers’ attention any items that were difficult to comprehend or which included words that were not clearly understood. Following the pilot, adjustments were made to optimize readability.

5. The extent of random sampling had to be estimated due to unanticipated logistical difficulties that undermined complete and accurate tracking of participation while simultaneously collecting data anonymously.

6. “Quality” in this context pertains to the degree to which a prison’s culture is value-driven and where staff embrace and demonstrate prosocial values and behaviors such as integrity, responsibility, or respect for people’s inherent worth or the valuing of community and productivity. In the context of the PF-PCA instrument’s Overall Culture scores, higher scores reflect higher quality.

7. The PF-WBI overall score is calculated as the average response value to a four-point response scale, corresponding to the following response anchors: False, Slightly True, Mostly True, Very True. PF-PCA subscale scores are the average response to their defining subsets of constituent items.

8. The assessment battery adminsistered to staff in this project included two extra assessment instruments that were not considered appropriate for administration to prisoners, based upon their design. This made participation lengthier and potentially more burdensome for staff participants.

9. Interested parties who would like more information about potential partnerships with PF, in relation to the PF-WBI, PF-PCA and/or the described web-hosted application, can inquire at: info@pfm.org. General information related to assessment tools developed by PF's research team are also accessible here: www.prisonfellowship.org/about/research-and-evaluation-library/
